# Hyperbaric Oxygen Therapy for Patients With Viral or Radiation-Induced Hemorrhagic Cystitis

**DOI:** 10.7759/cureus.80755

**Published:** 2025-03-18

**Authors:** Caiã Fraga Carvalho, Vincius C Lopes, Gabriel Agareno, Ana Clara Spessoto, Fernando Nestor Facio, Luís Cesar Fava Spessoto

**Affiliations:** 1 Urology, Faculty of Medicine of São José do Rio Preto (FAMERP), São José do Rio Preto, BRA; 2 Medicine, Medical School of Catanduva, Catanduva, BRA

**Keywords:** hematuria, hyperbaric oxygen therapy, radiation-induced bladder injury, radiotherapy, radiotherapy-inducted cystitis

## Abstract

Background: Hemorrhagic cystitis (HC) is characterized by diffuse inflammation and bleeding of the lining of the urinary bladder. This study investigated hyperbaric oxygen therapy (HBOT) in patients with viral or radiation-induced HC.

Methods: A retrospective analysis was performed involving 33 patients diagnosed with HC who received HBOT. Data analysis involved the Kruskal-Wallis test and Spearman’s correlation coefficients to determine the strength of correlations between variables.

Results: Of the 33 patients, nine (27.3%) had radiation-induced HC, and 24 (72.7%) had viral HC. Among those with viral HC, 12 (50%) tested positive for BK virus by polymerase chain reaction (PCR). HBOT was performed in a multiplace hyperbaric chamber at 2.5 absolute atmospheres (ATA) for 90 to 120 minutes, and the median number of sessions was 11. The median duration of treatment was 14 days, with 81.8% (n = 27) obtaining an improvement in macroscopic hematuria, 12.1% (n = 4) interrupting treatment, and 6.1% (n = 2) dying. HC did not recur in 57.6% of the sample (n = 19) in three years of follow-up. A significant correlation was found between the degree of hematuria and the number of sessions required (p = 0.0025). Radiation-induced HC was associated with higher degrees of hematuria (p = 0.007). A correlation was found between etiology and recurrence after the conclusion of treatment (p = 0.029).

Conclusion: Identifying the cause of HC and classifying the degree of hematuria are important to planning the number of HBOT sessions needed for an improvement in symptoms and a reduction in the rate of recurrence after treatment. The present findings suggest clinical benefits from HBOT in the treatment of HC.

## Introduction

Hemorrhagic cystitis (HC) is characterized by diffuse inflammation and bleeding of the lining of the urinary bladder. HC poses a challenge due to the diverse etiologies and degree of severity, ranging from mild hematuria (blood in the urine) to hemorrhage with the risk of death. Among the causes of the condition, viral infection and radiotherapy are the most common [1,2]. 

Viral HC is frequently associated with BK polyomavirus, John Cunningham (JC) virus, and cytomegalovirus, especially in immunosuppressed patients undergoing bone marrow transplantation or immunosuppressant therapy [2]. Radiation-induced HC is a late-onset complication of pelvic radiotherapy in patients treated for prostate or bladder cancer [3]. The harm caused by radiation leads to endarteritis and fibrosis, resulting in a reduction in vascularization and ulceration of the lining of the bladder [4]. 

Conventional management of HC includes intravesical treatments, such as irrigation with 0.9% saline solution, alum, electrocauterization, phenol, or formalin, and systemic medication, such as tranexamic acid or corticoids [5]. However, these approaches often have limited success, especially in severe cases, and invasive procedures may be required, such as urinary diversion or cystectomy [6].

Hyperbaric oxygen therapy (HBOT) is a promising adjunct treatment modality that improves the supply of oxygen to hypoxic tissues, promotes angiogenesis, reduces edema, and facilitates the healing process [7], making it a valuable therapy for HC. The present study investigated the results of HBOT in patients with viral or radiation-induced HC, considering the clinical characteristics of hospitalized patients and correlations between the etiology, degree of hematuria, number of HBOT sessions, and recurrence. 

## Materials and methods

A retrospective analysis was performed of 33 patients diagnosed with HC who received HBOT at the São José do Rio Preto Base Hospital in the state of São Paulo, Brazil, between 2013 and 2022. The study was based on a review of patient records available in the institutional electronic system. Incomplete records and cases of HC due to other etiologies were excluded from the analysis. This study received approval from the Human Research Ethics Committee of the São José do Rio Preto School of Medicine (FAMERP), Brazil (certificate number: 84874524.0.0000.5415).

Data were collected on demographic characteristics (sex and age), etiology (HC induced by BK virus confirmed by polymerase chain reaction (PCR) or radiation-induced HC), clinical characteristics (degree of hematuria classified as grades I to IV, number of HBOT sessions, and duration of treatment in days), aspects related to the outcome (improvement in hematuria, interruption of treatment, complications, and mortality), and recurrence of HC during a three-year follow-up period. 

All patients were treated by the same health team in the hospital setting. Patients underwent HBOT in a multiplace hyperbaric chamber at 2.5 absolute atmospheres (ATA) for 90 to 120 minutes. The number of sessions was determined based on the clinical response. 

Statistical analysis

Descriptive statistics were performed for demographic and clinical variables with the calculation of frequencies as well as measures of central tendency and dispersion. The Kolmogorov-Smirnov test was used to determine the distribution (normal or non-normal) of the data. Inferential analyses involved the Kruskal-Wallis test and the calculation of Spearman’s coefficients to determine the strength of correlations between variables. A p-value of ≤0.05 was considered indicative of statistical significance. The computation programs used were IBM SPSS Statistics for Windows, Version 23 (Released 2015; IBM Corp., Armonk, New York, United States) and PRISMA Version 6.10 (GraphPad Software Inc., San Diego, California, United States).

## Results

The sample was composed of 33 patients diagnosed with HC who received HBOT. The demographic and clinical characteristics of the patients are displayed in Table 1. Viral HC accounted for 72.7% of the cases (n = 24), and 50% of these patients (n = 12) tested positive for BK virus by PCR. Radiation-induced HC accounted for 27.3% of the cases (n = 9). The median age was 41 years (range: 15 to 80 years). The median age was 21 years among the patients with viral HC (range: 15 to 35 years) and 74 years among those with radiation-induced HC (range: 60 to 80 years). Most patients were males (72.7%; n = 24), and nine (27.3%) were females. 

**Table 1:** Demographic characteristics of patients with hemorrhagic cystitis (HC) n = number of individuals; M = male; F = female; age values correspond to median, minimum, and maximum; values between parentheses for sex correspond to percentage

Characteristic	Total (n = 33)	Viral HC (n = 24)	Radiation-induced HC (n = 9)
Age (years)	41 (15-80)	21 (15-35)	74 (60-80)
Sex			
M	24 (72.7)	18.0 (75)	6 (66.7)
F	9 (27.3)	6.0 (25)	3 (33.3)

At the beginning of treatment, the median degree of hematuria was classified as grade III. The median quantity of HBOT sessions required was 11 (range: 5 to 20), and the median duration of treatment was 14 days (range: 7 to 28 days). With regard to the clinical outcome, 81.8% (n = 27) of the patients experienced an improvement in hematuria. Interruption of treatment occurred in 12.1% of cases (n = 4) for unspecified reasons. No complications related to the use of HBOT were reported during the study period. The mortality rate was 6.1% (n = 2), and the deaths were not directly related to HBOT. HC did not recur in 57.6% of the sample (n = 19) in three years of follow-up, whereas HC recurred in 42.4% (n = 14).

Spearman’s coefficients (Table 2) demonstrated a significant positive correlation between the initial degree of hematuria and the number of HBOT sessions required (p = 0.0025). A significant correlation was found between etiology and the degree of hematuria, as radiation-induced HC was associated with higher degrees of hematuria (p = 0.007). No significant correlation was found between etiology and the number of HBOT sessions (p = 0.884). A significant difference between etiologies was observed in post-treatment recurrence (p = 0.029), with radiotherapy associated with a higher recurrence rate, suggesting that the etiology of HC influences the prognosis.

**Table 2:** Correlations between variables analyzed *statistical significance

Correlation	p
Degree of hematuria vs. number of sessions	0.0025*
Etiology vs. degree of hematuria	0.007*
Etiology vs. number of sessions	0.884
Etiology vs. posttreatment recurrence	0.029*

## Discussion

HC, particularly when associated with radiotherapy, is a complication that is difficult to manage due to the multifactorial physiopathology, which includes endothelial damage, progressive arteriopathy, and tissue fibrosis, resulting in chronic hypoxia of the urinary bladder tissue [1]. The present results confirm the effectiveness of HBOT as an adjunct therapeutic approach, as demonstrated by the significant reduction in hematuria, which is in agreement with studies found in the literature reporting success rates higher than 75% regarding the resolution of urinary signs in patients with postradiation HC [6].

HBOT was initially introduced for the treatment of radiation-induced HC, demonstrating efficacy and few adverse effects [8]. In 1985, Weiss et al. [9] reported the effectiveness of HBOT in treating radiation-induced HC. Since then, the treatment of this condition with HBOT has been widely used. HBOT not only improved symptoms but also contributed to the tissue regeneration process, promoting angiogenesis and the deposition of fibroblasts in injured tissue [10,11].

The positive correlation between greater hematuria severity and the number of HBOT sessions suggests the need for an individualized approach, with the adaptation of the protocol to the degree of bladder injury. Response to HBOT has also been correlated to the initial severity of hematuria. Mougin et al. demonstrate that hematuria grade <3 was associated with a successful therapeutic outcome [12]. Moreover, the higher degree of hematuria and greater recurrence of HC in patients having been submitted to radiotherapy compared to cases of a viral etiology reflects the cumulative impact of radiotherapy on the microvasculature of the urinary bladder.

The safety of HBOT is widely documented in the literature, with a low incidence of side effects including middle ear barotrauma, sinus and paranasal sinus barotrauma, ocular side effects, hypoglycemia, oxygen-induced seizures, and claustrophobia [13]; the most common of which is ear pain occurring in up to 33.3% of patients [14,15]. In this investigation, no side effects were found in the studied patients. This tolerability makes HBOT an attractive option even for older patients and those with multiple comorbidities. Thus, the present study confirms the potential of HBOT in the satisfactory management of HC, especially in refractory cases. However, the lack of prospective randomized clinical trials limits the standardization of the method. Future studies should focus on the definition of the ideal duration of treatment and the identification of response predictors that can contribute to expanding the implementation of HBOT in clinical practice. This study was conducted at a single center with a small sample size and a retrospective design, which limits the generalization of the data.

## Conclusions

HBOT seems to be a safe and effective adjunct therapy for the treatment of viral and radiation-induced HC, leading to an improvement in hematuria and a reduction in refractiveness. Identifying the cause of HC and classifying the degree of hematuria are important to personalizing treatment and predicting outcomes.
